# Automated Cryo‐EM and Supervised Machine Learning Enable Reproducible Characterization of Extracellular Vesicles and Co‐Isolating Particles

**DOI:** 10.1002/jev2.70273

**Published:** 2026-04-14

**Authors:** Agustin Enciso‐Martinez, Frank G. A. Faas, Anja W. M. de Jong, Ton G. van Leeuwen, Rienk Nieuwland, Edwin van der Pol, Peter ten Dijke, Roman I. Koning

**Affiliations:** ^1^ Oncode Institute and Department of Cell and Chemical Biology Leiden University Medical Center Leiden The Netherlands; ^2^ Biomedical Engineering & Physics; Amsterdam Cardiovascular Sciences, Cancer Center Amsterdam Amsterdam UMC, University of Amsterdam Amsterdam The Netherlands; ^3^ Laboratory of Experimental Clinical Chemistry Laboratory Specialized Diagnostics & Research Department of Laboratory Medicine Amsterdam UMC, University of Amsterdam Amsterdam The Netherlands; ^4^ Electron Microscopy Facility, Department of Cell and Chemical Biology Leiden University Medical Center Leiden The Netherlands

**Keywords:** automation, cryo‐electron microscopy, extracellular vesicles, lipoproteins, machine learning, particle detection, artificial intelligence

## Abstract

Cryo‐electron microscopy (cryo‐EM) is the only technique capable of visualizing the lipid bilayer of extracellular vesicles (EVs), enabling their distinction from non‐EV particles. However, the application of cryo‐EM for EV sample characterization has been limited by a combination of low imaging throughput and complex image analysis of the structurally diverse EVs. To address these challenges, we developed a workflow combining automated cryo‐EM image acquisition with supervised machine learning (sML)‐assisted particle detection and classification. Automated image acquisition facilitates the routine acquisition of thousands of cryo‐EM images with consistent quality, enabling the imaging of hundreds of EVs. sML‐assisted particle detection enabled efficient and reproducible identification, size measurement, and structural classification of EVs. Furthermore, using sML we are able to differentiate EVs from non‐EV particles, such as lipoproteins and protein aggregates, which might co‐isolate due to overlapping physical properties or by physical association with EVs. In mixed EV‐lipoprotein samples, we demonstrate that our pipeline can distinguish EVs and differentiate between high‐density (HDL), low‐density (LDL), and very low‐density (VLDL) lipoproteins. Our automated cryo‐EM and sML workflow overcomes key limitations of EV characterization using cryo‐EM by increasing imaging throughput and enabling reproducible EV characterization. Furthermore, this method provides a tool for analysing EV heterogeneity, sample purity, and co‐isolated contaminants, advancing the field of EV research.

## Graphical abstract

Automated cryo‐electron microscopy enables high‐throughput and unbiased image acquisition, which, combined with supervised machine learning allows modelling and detection of lipid membranes and other structures, like lipoproteins. This integration provides a powerful approach for analysis of EV size and morphology, as well as sample purity assessment.

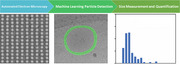

**Abbreviations**
2D: two dimensional
3D: three dimensional
ATTC: American tissue type collection
CCD: charge coupled device
CNN: convolutional neuronal network
Cryo‐CLEM: cryo‐correlative light and electron microscopy
Cryo‐EM: cryo‐electron microscopy
Cryo‐ET: cryo‐electron tomography
DED: direct electron detector
DNA: deoxyribonucleic acid
EDTA: ethylenediaminetetraacetic acid
EPU: E Pluribus Unum
EV: extracellular vesicle
HDL: high density lipoprotein
ISEV: International Society of Extracellular Vesicles
LD: lipid droplet
LDL: low density lipoprotein
MDA‐MB: MD Anderson ‐ metastatic breast
MISEV: minimal information for studies for extracellular vesicles
RNA: ribonucleic acid
ROI: region of interest
SD: standard deviation
SDS: sodium dodecyl sulphate
SEC: size exclusion chromatography
SEM: scanning electron microscopy
sML: supervised machine learning
SPA: single particle analysis
TEM: transmission electron microscopy
TFF: tangential flow filtration
UF: ultrafiltration
VLDL: very low density lipoprotein

## 1 Introduction

Extracellular vesicles (EVs) are nano‐ to microscale particles, enclosed by a lipid bilayer and released by virtually all cell types. EVs play key roles in cell waste removal and intercellular communication, and have emerged as promising biomarkers and therapeutic agents (Buzas 2023; van Niel et al. 2018). They originate from distinct cellular pathways, including the endosomal system (exosomes), outward budding of the plasma membrane (microvesicles or ectosomes), and programmed cell death (apoptotic bodies). While EVs comprise a highly heterogeneous population carrying diverse molecular cargo, their occurrence is remarkably conserved across species, underscoring their fundamental biological importance in physiology and disease. Studying their structure and function is therefore critical to understanding their biological roles and potential diagnostic and therapeutic applications.

According to the MISEV guidelines, EVs are defined as lipid bilayer‐delimited particles, which are released by cells and cannot replicate (Welsh et al. 2024). Direct visualization of this bilayer is therefore critical for EV identification and for distinguishing EVs from non‐vesicular particles of overlapping size, such as lipoproteins, protein aggregates and other impurities. These particles are commonly present in (crude) EV isolates, and may arise from co‐isolation due to overlapping physical properties or from physiological association with the EV surface as part of a biomolecular corona, potentially contributing to EV function (Busatto et al. 2020; Radeghieri et al. 2022; Sódar et al. 2016). Contaminations may lead to confounding bioactivity in mechanistic studies in which effects are wrongly attributed to EVs and compromise the reproducibility and specificity of EV research. Moreover, lack of standardization for validating contaminant removal in diagnostic and therapeutic EV preparations may impede (multi‐centre) clinical validation.

Cryo‐electron microscopy (cryo‐EM) uniquely enables visualization of the lipid bilayer of individual EVs in their native hydrated state, while simultaneously providing information on particle size, morphology, and structural details (Welsh et al. 2024; Szatanek et al. 2017; Yuana et al. 2013; Poliakov et al. 2009). As such, cryo‐EM not only supports rigorous EV identification and sample quality and purity assessment, but also offers insights into structural heterogeneity that may be relevant for EV biogenesis, function, and downstream biological interpretation.

Although cryo‐EM provides valuable information about EV‐containing samples (Welsh et al. 2024), its broader application in the EV field is hindered by several challenges: (i) limited accessibility of the technique, (ii) low imaging and analysis throughput (Yuana et al. 2013), and (iii) potential user‐dependent bias in region‐of‐interest (ROI) selection (Rikkert et al. 2019). Traditionally, manual acquisition of a limited number of images has been the standard approach in (cryo‐)EM (Arraud et al. 2014; Brisson et al. 2017), with some exceptions (Emelyanov et al. 2020; Hoog and Lotvall 2015; Parra et al. 2024). However, manual image acquisition is time‐consuming, limiting the number of particles that can be analysed, and subjective ROI selection may lead to irreproducible results (Rikkert et al. 2019). Manual cryo‐EM approaches also limit the comprehensive characterization of EVs and other sub‐micrometre particles, posing a significant limitation when concentrations are low (e.g., < ∼10^6^ particles/mL). While EVs can be concentrated, such methods often induce particle aggregation and structural alterations, compromising EV morphology and integrity, both of which are crucial for structural classification (Cocozza et al. 2020). To mitigate this issue, gentle concentration and purification techniques are recommended to preserve EV structure and prevent aggregation. However, such techniques typically result in low EV concentrations, making large‐scale imaging necessary and analysis challenging. Hence, while non‐automated methods can confirm the presence of EVs, they fail to provide an objective, observer‐independent representation of the sample composition or an accurate assessment of the sample quality.

Isolation of EVs from conditioned media or bodily fluids (e.g., blood, milk, urine) typically involves size‐, density‐, solubility‐, or immunoaffinity‐based methods, which balance yield and purity. While established EV analysis methods, including marker‐based assays (e.g., immunofluorescence, flow cytometry) and omics‐based analyses, provide information on EV‐associated molecules, they do not directly visualize particle morphology. As such, these methods may not distinguish EVs from co‐isolated soluble proteins or other non‐EV particles.

A major advantage of imaging isolated EV samples with cryo‐EM, beyond direct visualization of the EV lipid bilayer, is the ability to directly observe co‐isolated particles, contaminants, and EV associated structures. Typical non‐EV particles, such as lipoproteins (HDL, LDL, VLDL, and chylomicrons), protein complexes (supermeres and exomeres) or aggregates, viruses, and smaller structures including RNA and DNA, can be directly detected based on their structural features. Many of these entities may remain undetected without targeted labelling or may be incorrectly classified as EVs when using other analytical techniques. Failure to identify such co‐isolated components can result in incomplete sample characterization and irreproducible results when samples are used in functional studies.

Integrating cryo‐EM with the commonly used analysis techniques offers direct structural insights into EV preparations, revealing sample heterogeneity and the presence of morphologically distinct contaminants. Here, we introduce a workflow combining automated cryo‐EM imaging with supervised machine learning (sML) to detect and classify EVs and non‐EV particles, even in low‐concentration (< ∼10^6^ particles/mL) samples, offering a complementary approach that improves sample characterization and supports robust downstream analyses.

While manual segmentation, classification and size measurement can be effective for EV analysis of small datasets with a few hundred images, these approaches become inefficient and less reproducible as dataset size and sample complexity increase. To address this limitation, this study combined automated cryo‐EM image acquisition and analysis using sML to characterize EVs and other submicron particles. Automated cryo‐EM image acquisition enabled operator‐independent and reproducible imaging, providing direct visualization of lipid bilayer‐delimited EVs alongside non‐EV particles, such as lipoproteins. sML particle detection enabled the classification of EVs and non‐EV particles, while Hough circle analysis allowed the automated quantification of EV numbers and sizes, ensuring reproducible particle analysis. By applying these workflows, we showed that EV‐enriched samples from cultured MDA‐MB‐231 breast cancer cells contain structurally different EV classes, as well as small protein aggregates resembling exomeres. In addition, EVs were reliably distinguished from co‐isolated particles such as lipoproteins, both in defined EV–lipoprotein mixtures and in human plasma samples after removal of soluble proteins. Finally, we argue that comprehensive and reproducible analysis of purified EV samples using cryo‐EM imaging and image analysis facilitates unbiased analysis of EV‐containing samples.

## 2 Materials and Methods

### 2.1 Workflow

**FIGURE 1:**
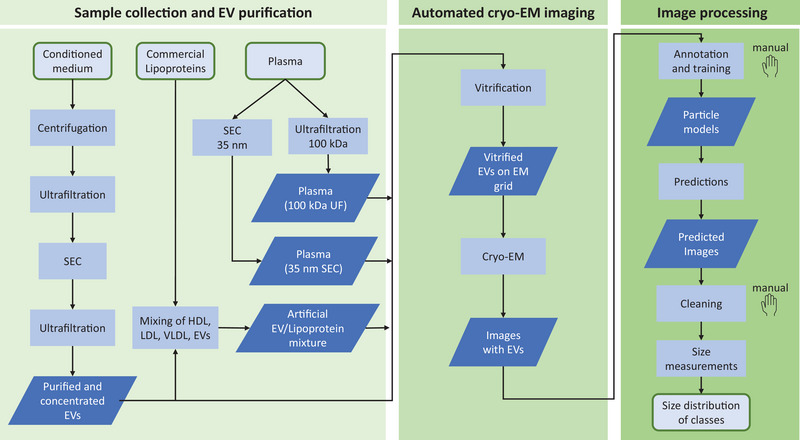
Overview of sample preparation, imaging and extracellular vesicle (EV) detection workflow. Schematic workflow chart for (i) sample collection and EV purification, including preparation of plasma and sample mixing, (ii) automated cryo‐electron microscopy (cryo‐EM) imaging, and (iii) image processing for automated classification and size distribution determination of EVs. Manual steps are denoted by a hand symbol.

Figure 1 shows the workflow for the analysis of EV‐containing samples. This workflow consists of: (i) Sample collection and EV purification, (ii) automated cryo‐EM imaging, and (iii) image processing, consisting of particle annotation and training, prediction, cleaning and size measurement. We isolated EVs from MDA‐MB‐231 human breast cancer cell‐conditioned medium to develop and validate our workflow. This cell line was chosen as it is used in many cancer biology‐oriented studies, and it is known to release EVs that promote cancer progression (González‐Callejo et al. 2023; Teixeira et al. 2023; Xie, Zhou, Li et al. 2022, Xie, Zhou, Su et al. 2022). To preserve the structural integrity of EVs, we used mild purification and concentration methods, that is, low speed centrifugation to remove cellular debris, ultrafiltration (UF) and size‐exclusion chromatography (SEC) to isolate EVs. We did not use ultracentrifugation to avoid potential aggregation of EVs and soluble proteins, and to minimize the risk of EV rupture associated with high g‐force pelleting (Linares et al. 2015). EV‐enriched samples were cryo‐fixed by vitrification to preserve the structure of EVs and enable cryo‐EM imaging (Yuana et al. 2013; Arraud et al. 2014). We collected cryo‐EM images of EVs using an automated data acquisition scheme, as used in single particle analysis (SPA) cryo‐EM, albeit at lower magnification than generally used (Thompson et al. 2019). This approach enables automated and reproducible acquisition of several hundreds of images per hour. Subsequent subjection to automated and reproducible particle detection algorithms allows for the comprehensive detection of particles in purified samples with low concentration (< ∼10^6^ particles/mL). This empowers the generation of image atlases of individual EVs, morphological classification of particles, and reliable particle size distribution measurements.

### 2.2 Cell Culture and EV Collection

Human breast cancer MDA‐MB‐231cell‐derived EVs were used as a test sample for cryo‐EM analysis. The MDA‐MB‐231 cell line has been extensively used in EV studies due to its aggressive metastatic properties and its ability to produce a high yield of EVs, making it an ideal model for investigating EV biology (Hoshino et al. 2015; Leal‐Orta et al. 2022; Campos et al. 2018; Tian et al. 2021; González‐King et al. 2022; Rontogianni et al. 2019). MDA‐MB‐231 (RRID:CVCL_0062) cells were cultured by seeding 3–5 × 10^5^ cells per T175 flask (Cellstar Grenier, 660175) in Dulbecco's Modified Eagle Medium (DMEM, Gibco, 41966) supplemented with 10% (v/v) foetal bovine serum (FBS) and 0.2% penicillin‐streptomycat 37°C in a humidified atmosphere containing 5% CO_2_. A total of 10–12 T175 flasks were used and the conditioned medium was refreshed twice per week. At 70% confluence, cells were washed three times with pre‐warmed phosphate‐buffered solution (PBS) and cultured in FBS‐free DMEM, supplemented with 0.2% penicillin‐streptomycin, to remove EVs or non‐EV particles from the FBS. After 48 h of incubation at 37°C and 5% CO_2_, the conditioned medium was collected in 50 mL tubes (Cellstar Grenier, 227261). At collection, the concentration of cells was ∼1 × 10^6^ cells/mL, and cell viability was >95% (Trypan blue assay). For a typical cryo‐EM preparation, 300–350 mL of conditioned medium was used. Cells were tested monthly for the absence of mycoplasma, and the cell line was authenticated by short tandem repeat profiling.

### 2.3 EV Purification

Conditioned medium in 50 mL tubes (Cellstar Greiner, 227261) was centrifuged (Eppendorf 5810R, RRID:SCR_019855) at 300 × *g* for 5 min at 4°C to remove floating cells. The supernatant was collected in new 50 mL tubes and centrifuged at 2000 × *g* for 15 min at 4°C to remove large particles. Next, ∼300 mL of supernatant was collected and concentrated using a peristaltic pump (Masterflex^TM^ L/S^TM^, RRID:SCR_019028) at 20 mL/min and tangential flow filtration (TFF‐Easy, Hansa Biomed) or UF (MWCO 100 kDa; AmiconUltra‐15 centrifugal filters, UFC9100, Merck Millipore Ltd.) at 3200 × *g* until a final volume of 1–2 mL was reached. The presence of EVs was assessed by immunoblotting for canonical EV tetraspanin markers, that is, CD9 and CD63 (Supplementary data 1 and 2). Next, SEC was performed using Izon's Automatic Fraction Collector to separate EVs from soluble components, according to the manufacturer's protocol. In brief, 500 µL of the concentrated EV‐containing sample was added to a 35‐nm SEC column (qEVOriginal, Gen 2, Izon Science, New Zealand), and after 2.9 mL of flowthrough, 4 EV‐containing fractions of 400 µL each were collected. Dulbecco's Phosphate Buffered Saline (DPBS, Corning Dulbecco's Phosphate‐Buffered Saline, #21‐031‐CV) was used as elution buffer. EV fractions were pooled and concentrated further to 50–250 µL by UF (MWCO 100 kDa; EVspinner, Hansa Biomed or AmiconUltra‐15 centrifugal filters, UFC9100, Merck Millipore Ltd.).

### 2.4 Lipoproteins and EV Mixtures

Purified human lipoproteins, namely, high‐density lipoproteins (HDL, 14.1 mg/mL, Cat. No.: LP3‐5MG, EMD Millipore Corp.), low‐density lipoproteins (LDL, 6.75 mg/mL, Cat. No.: 437644‐10MG, EMD Millipore Corp.), and very‐low‐density lipoproteins (VLDL, Cat. No.: 437647‐5MG, EMD Millipore Corp. (1.0 mg/mL) and Calbiochem (0.8 mg/mL)) were purchased and stored according to manufacturer's instructions. The purity of the lipoproteins was verified to be ≥95% pure (of total lipoprotein content) by electrophoresis (LDL and VLDL) or sodium dodecyl sulphate polyacrylamide gel electrophoresis (HDL) as specified by the manufacturer. To biochemically confirm the presence and identity of lipoproteins in the commercial preparations, immunoblotting was performed using antibodies against apolipoprotein A‐I and apolipoprotein B100 (Supplementary data 3 and 4). The different lipoprotein samples were further inspected by cryo‐EM after diluting the stock solutions tenfold in DPBS (Supplementary data 5, 6 and 7).

A lipoprotein mixture was prepared by mixing HDL, LDL and VLDL, each diluted tenfold in DPBS. Next, an EV‐lipoprotein mixture was prepared by combining the lipoprotein mixture with purified MDA‐MB‐231 EVs (isolated as described in *EV purification*) at a 1:10 EV‐to‐lipoprotein volume ratio. The EV‐lipoprotein mixture was incubated for 5 min at room temperature, and subsequently stored at 4°C for ~1 h prior to cryo‐fixation.

### 2.5 Plasma Collection and Processing

Blood of healthy donors was collected via the Leiden University Medical Center healthy voluntary donor service (LuVDS). All donors gave broad consent. The biomaterial and associated clinical data of all donors collected in the LuVDS are released for research purposes only, after being approved by the internal review board (BB23.006). Peripheral blood samples were collected from using Ethylenediaminetetraacetic acid (EDTA) tubes (Vacutainer, Becton Dickinson, USA). The first ∼3 mL of whole blood were discarded. The subsequent blood volume was centrifuged twice at 2500 × *g* for 15 min at 20°C. All samples were centrifuged without break, and the plasma fractions were carefully collected from approximately 10 mm above the buffy coat or cell pellet to minimize platelet. Next, plasma from different donors was pooled and filtered using an 800‐nm filter (Isopore, ATTP02500, Merck Milipore) to remove remaining platelets. Plasma samples were aliquoted, snap‐frozen in liquid N_2_ and stored at –80°C until use.

#### 2.5.1 Size Exclusion Chromatography (SEC)

Pooled plasma from 8 healthy donors was thawed in a 37°C water bath until only a small piece of ice remained (typically <1 min). SEC was performed using the Izon fraction collector and 35‐nm SEC columns (qEVoriginal Gen 2, Izon Science, New Zealand). A volume of 0.5 mL of thawed plasma was loaded onto the column, followed by Dulbecco's phosphate buffered saline (DPBS) as running buffer. After collecting an initial 2.9 mL flow‐through, 4 subsequent 0.4 mL fractions were collected and kept at 4°C. Fraction 2, corresponding to the expected EV‐enriched fraction, was selected for cryo‐EM preparation.

#### 2.5.2 Ultrafiltration (UF)

Pooled plasma from 4 healthy donors was thawed in a 37°C water bath until only a small piece of ice remained (typically <1 min). Next, 1 mL plasma was diluted 4x in DPBS and loaded onto a centrifugal filter (MWCO 100 kDa; Amicon Ultra‐4, UFC810024, Merck Millipore Ltd.). The filter was centrifuged at 3200 × *g* for multiple cycles up to a total time of 55 min and DPBS added in between. The retentate was then prepared for cryo‐EM.

### 2.6 Sample Preparation for Cryo‐EM

Samples for cryo‐EM were prepared by plunge‐freezing of a thin (∼200 nm) layer of liquid sample on a holey carbon film, which was supported by a cupper (EM) grid, into a cryogen coolant. During this process the thin sample layer was cooled at such high rates (> 10.000°C/s) that the sample transforms into a thin vitreous, non‐crystalline, electron‐transparent, water layer in which the isolated EVs were perfectly preserved. In practice, Quantifoil 300 Mesh cupper grids with 2/2 or 2/1 (hole size, hole spacing) carbon foil (Quantifoil micro tools, GmbH) were glow discharged in air at 0.2 mbar, 25 mA and 30 s using a PELCO easiGlow^TM^ (Ted Pella, Inc.) to render the carbon layer hydrophilic. Onto the glow discharged grids, 3 µL of sample was added and subsequently blotted away for 3 s using filter paper (Whatman no. 4) at 85%–95% humidity and room temperature using a Leica EM GP (electron microscopy grid plunger, Leica Microsystems) to generate a stable and thin water layer. The grid was subsequently plunged into liquid ethane at −182°C or liquid ethane/propane (2/1; v/v) at −196°C (Resch et al. 2011) to generate the vitreous water layer. Grids were clipped into autoclip rings for use in the multigrid autoloader system for the Arctica microscope and stored under liquid nitrogen until further use.

### 2.7 Automated Cryo‐EM Imaging

Two‐dimensional cryo‐EM imaging was performed on a Talos Arctica (Thermo Fisher Scientific). Data collection was operated by EPU (E Pluribus Unum) software (Thermo Fisher Scientific) in multi‐grid mode. This software enables automated imaging after first recording a grid overview and selection of the imaging positions. All settings can be stored and reused to ensure reproducible imaging conditions. Imaging speed and reproducibility were optimized by using (i) a single preset for the imaging conditions, (ii) multi‐sample image acquisition per microscope session, and (iii) fast image‐beam shift to image different positions, instead of stage movements. Using software for automated image acquisition (EPU) several hundreds of images per hour can be recorded, either from a single sample or distributed across multiple samples. Additionally, acquiring large image datasets allowed assessment of EV heterogeneity and purity of the sample.

The following magnification setup was used: atlas 110x, hole 6300x, data 15000x, focus 49000x. (see Supplementary data for EPU settings file, which can be used on other systems running EPU). EM grid overviews were made at 110x magnification. In this overview, around ten grid squares per sample were manually selected for data acquisition. Images were recorded in counting mode and zero loss peak (ZLP) imaging in movie mode on a K3 direct electron detector (Gatan). A magnification of 15,000x (corresponding to a pixel size of 0.55 nm at specimen level) was used to image one full 2 µm hole per image. A defocus value of −5 µm, and an electron dose of ∼4 e/Å^2^/s with 8 s exposure time (corresponding to a total dose of ∼32 e/Å^2^) were used at this magnification, to optimize the contrast between the individual leaflets of the lipid bilayer (at ∼4 nm distance).

Images were collected either on a direct electron detector (DED) in movie mode or on a fibre‐coupled scintillator charged coupled device (CCD) camera. A DED has a high detection quantum efficiency (DQE) and therefore is more sensitive than a scintillator camera. A DED records movies at ∼1500 fps and can detect single electrons. Frames can be aligned to correct for sample movements, producing images with the highest quality currently available, which are typically used in low‐dose and SPA cryo‐EM imaging. Movies were aligned using MotionCorr2 (Zheng et al. 2017) and converted to tiff using EMAN2 (Tang et al. 2007) (e2proc2d.py *.mrc @.tif ‐outmode uint8 ‐fixintscaling sane). For detection of lipoproteins, images were bandpass filtered using FIJI (Schindelin et al. 2012) (“Bandpass Filter Filter_Large = 50, Filter_Small = 2 suppress = none tolerance = 5”). Alternatively, a Ceta camera (Thermo Fisher Scientific) was used with the following magnification setups: atlas 110x, hole 6300x, data 22000x, focus 49000x. Data was recorded at a magnification of 22000x, corresponding to a pixel size of 0.65 nm at specimen level, a defocus of −4 µm, and an electron dose of ∼35 e/Å^2^/s with 1 s exposure time. Images acquired using a CCD are single images, recorded at higher electron flux and shorter exposure times, which do not need any postprocessing by movie alignment correction. CCD images are of lower quality than DED images.

### 2.8 Image Processing

For sML‐assisted analysis, images were annotated, models were trained and images were segmented using a custom user interface. In short (for a detailed protocol, see Supplementary data), images were stitched into a composite pyramid image, desired structures were manually annotated in the images, and ROI boxes were placed to extract and generate ground truth training set data. Model parameters were calculated in Keras (Chollet et al. 2015) and TensorFlow (Abadi et al. 2015) using an adapted version (manuscript in preparation) of a pre‐existing convolutional neural network (Xiao et al. 2018). Predicted datasets were cleaned for quantification and size measurements by manually removing obvious false positives (e.g. carbon hole edges or ice contamination) and occasionally filling in incomplete particle predictions. Surface areas from every particle (also oval‐shaped particles) were exported and converted to a particle diameter (d = 2(A/π)^1/2^).

The Hough circle transform plugin from UCB vision sciences in Fiji was used for automated analysis of particle sizes. In short (for a detailed protocol, see Supplementary data), predicted segmentations at 4x binning (at 2.2 nm pixel size) were extracted from the pyramid images, and the composite image was transformed back into an image stack. The image stack was de‐speckled to remove small false positive pixels. Edge detection was used to generate outlines of the circles from the particle segmentations, and skeletonization was used to outline rings from the bilayer segmentations. Hough transforms were applied to the bilayer segmentations, resulting in a particle size distribution table. Circles and rings with a radius between 6 and 100 pixels (26.4 and 440 nm) were detected above a Hough threshold score of 0.8 (circles) or 0.5 (rings).

## 3 Results

### 3.1 Automated Detection and Quantification of EVs and Other Particles in Large Datasets

**FIGURE 2:**
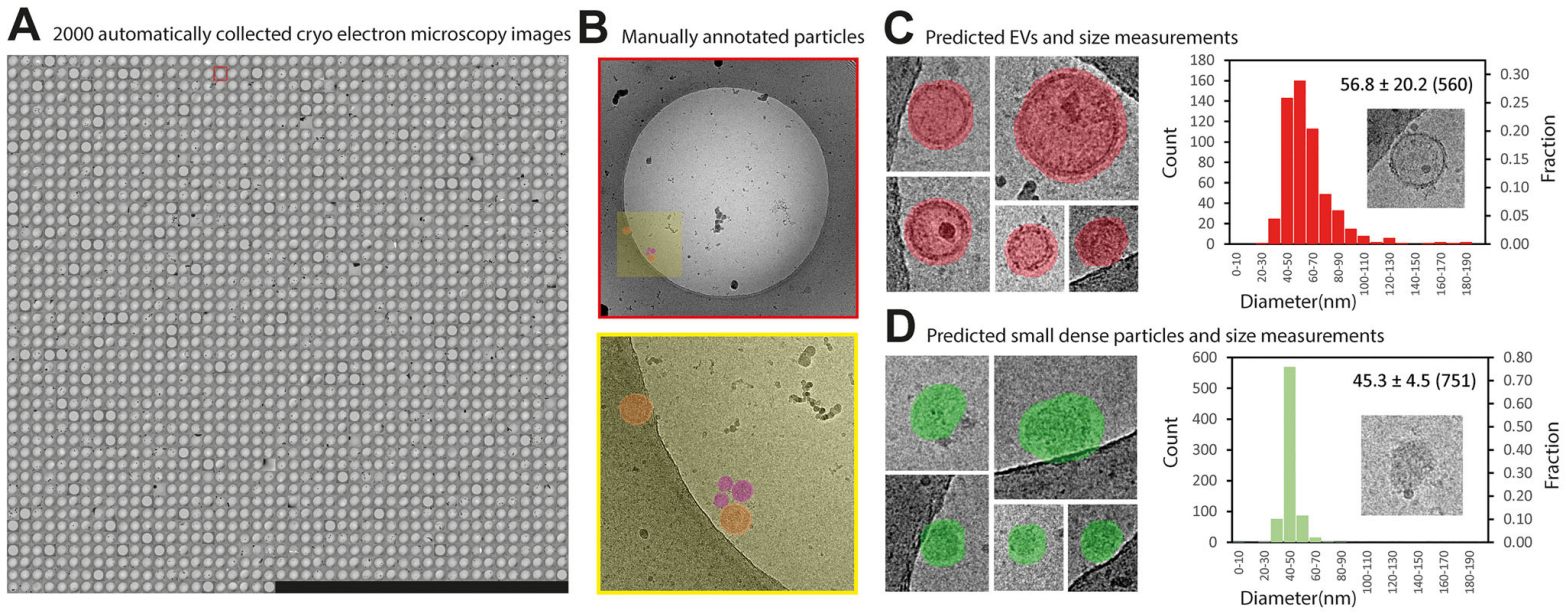
Automated data collection and supervised machine learning detection of different particle classes and size distribution analysis of MDA‐MB‐231 breast cancer cell derived particles. (A) Collage of 2000 automatically acquired cryo‐EM images targeting 2 µm diameter holes inside carbon support foil. (B) Top: cryo‐EM image of the single hole with extraction box (yellow). Bottom: enlarged box with manually annotated EVs orange) and EV‐like particles (purple). (C) Examples of machine learning predicted EVs (red), size distribution measurements and median diameter derived from predicted particle surfaces, standard deviation and particle count. (D) Examples of predicted small dense particles, resembling exomeres (green), their size distribution and median diameter derived from predicted particle surface, standard deviation and particle count.

For the development and validation of an automated workflow, we used a sample containing MDA‐MB‐231‐derived EVs and automatically acquired 2000 images on a CCD camera (Figure 2A). Next, we applied a sML approach for particle segmentation, in combination with automated particle quantification and size calculation. To train our segmentation models, we manually annotated subsets of two main classes that were present in the sample: (i) EVs, that is, particles enclosed by a lipid bilayer, and (ii) small (∼45 nm) dense particles that were not delimited by a lipid bilayer (Figure 2B), which resemble exomeres (Yu et al. 2025). We then trained a model for each class using an adapted convolutional neural network (CNN) (Dzyubachyk et al. 2021) that was initially used to detect mitochondria in 3D datasets (Xiao et al. 2018). EV and small dense particle image segmentations were predicted using these models. Size measurements were performed by extracting the surface area from the particle segmentations and computing the average particle diameters based on these measurements. The small dense particles, with a median diameter of 45.3 nm (SD = 4.5 nm, *n* = 751), outnumbered the EVs, which had a median diameter of 56.8 nm (SD = 20.2 nm, *n* = 560) (Figures 2C,D).

For generating the EV model that was used for the predictions of EVs in Figure 2, 181 ROIs were used (0.57% of the total image area), which contained 161 EVs (29% of the number of total number of predicted EVs). For generating the small dense particle model 416 ROIs were used (1.3% of the total image area), which contained 202 small dense particles (27% of the number of predicted particles). Model performance was estimated by visually inspecting 4.5% of the dataset, yielding a precision of 83% and an overall accuracy of 85% for EV detection, and a precision of 91% with an accuracy of 66% for detection of small dense particles.

### 3.2 Automated Detection of EVs Using a CNN Trained on Bilayer Recognition

**FIGURE 3:**
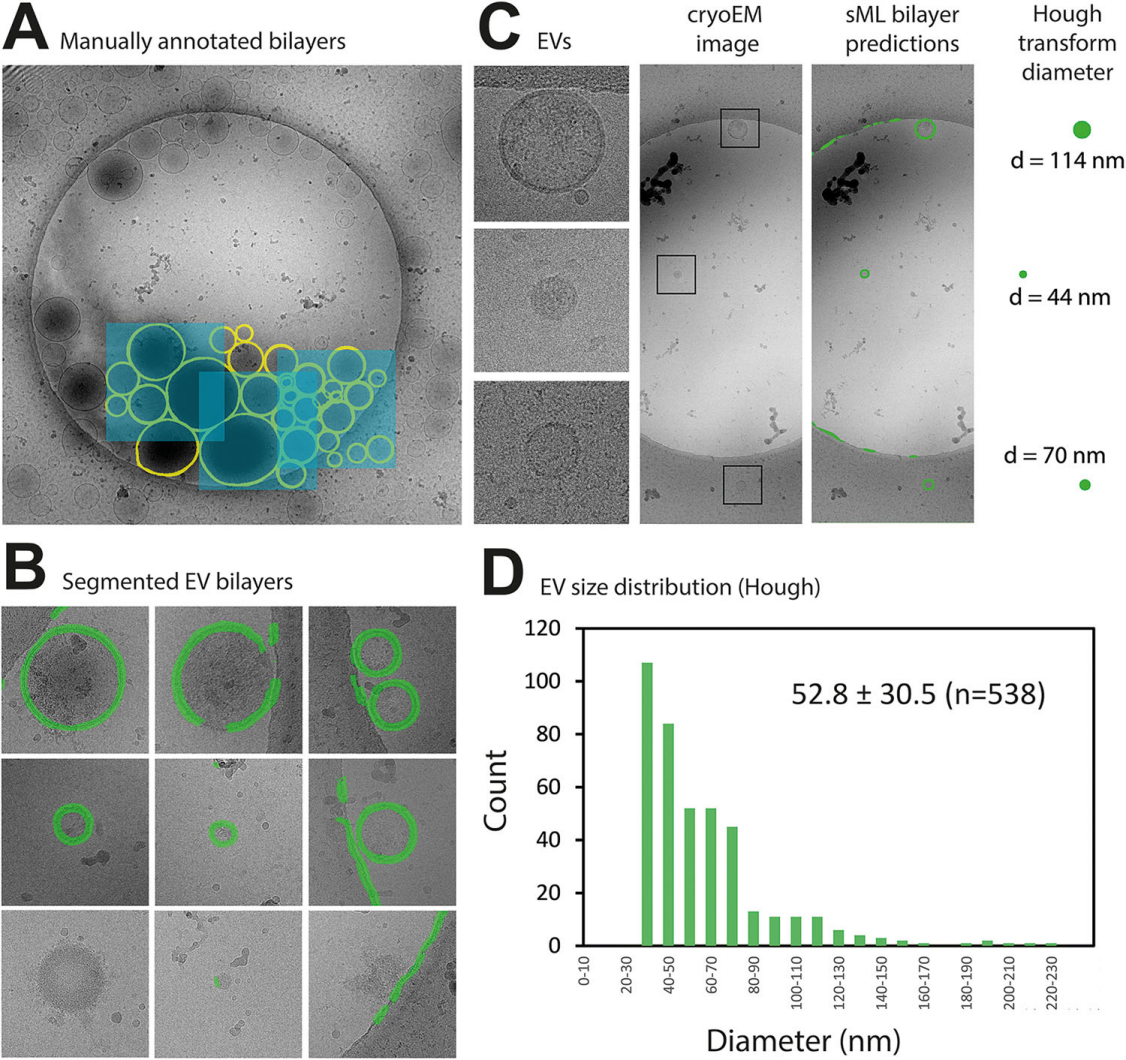
Automated EV lipid bilayer detection and particle size distribution measurements using a combination of sML and Hough transform circle detection. (A) Manual annotation of lipid bilayers (yellow) in liposome samples and boxes (blue) for model training. (B) Predictions from the bilayer model show that EVs are segmented (green), while other particles (bottom row, e.g., VLDL) are not detected. (C) Three EVs (left panels) of which the bilayers are detected (green, third panel), and diameters are automatically extracted by Hough circle transform and centroid detection (right panel). (D) Size distribution of MDA‐MB‐231 cell‐derived EVs and median diameter, standard deviation and count.

Our initial particle annotations, models and predictions were based on whole EVs and small dense particles. Since larger EVs were underrepresented in the images, and therefore also in the annotations and the model, large EVs were less or only partially predicted by the EV model, making both quantification and surface based size measurements inaccurate. Also, lipid bilayers were not always well visible in the CCD images at the imaging condition that we used. To increase image quality and particle detection accuracy, we pursued to identify EVs based on the presence of a lipid bilayer in images recorded using a DED in movie mode after frame alignment. Therefore, we developed a CNN model to enable the network to detect the lipid bilayer, since the presence of a bilayer is a defining feature of EVs (Welsh et al. 2024). This model was trained on concentrated liposome samples, which provided an abundance of bilayers (Figure 3A). The lipid bilayer models identified EVs with a precision of 90% and an accuracy of 89% (determined from 125 of the 1138 images). VLDL contaminants and small amorphous particles lacking a lipid bilayer were not detected by the model, In contrast, carbon foil edges were frequently misclassified as false positive predictions (Figure 3B, bottom row). These artifacts were automatically excluded from particle size measurements using Hough circle transforms. Since lipid bilayer detection of EVs results in ring‐like segmentations, particle diameters cannot be deduced from the surface area measurements. To address this, we employed Hough circle transformation to the predicted EV bilayer segmentations to accurately determine the EV diameters (Figure 3C). In our test sample, the median EV diameter was 52.8 nm (SD = 30.5 nm, *n* = 538). A key advantage of this approach is its ability to detect incomplete circles, enabling diameter estimation even when the EV bilayer is only partially predicted. Additionally, this method minimizes the inclusion of non‐circular false positives. Thus, combining sML for bilayer segmentation with Hough circle transformation resulted in a robust and automatic workflow for EV detection and size measurement (Figure 3D).

### 3.3 Automated Detection of EVs and Lipoproteins in Heterogeneous Samples

**FIGURE 4:**
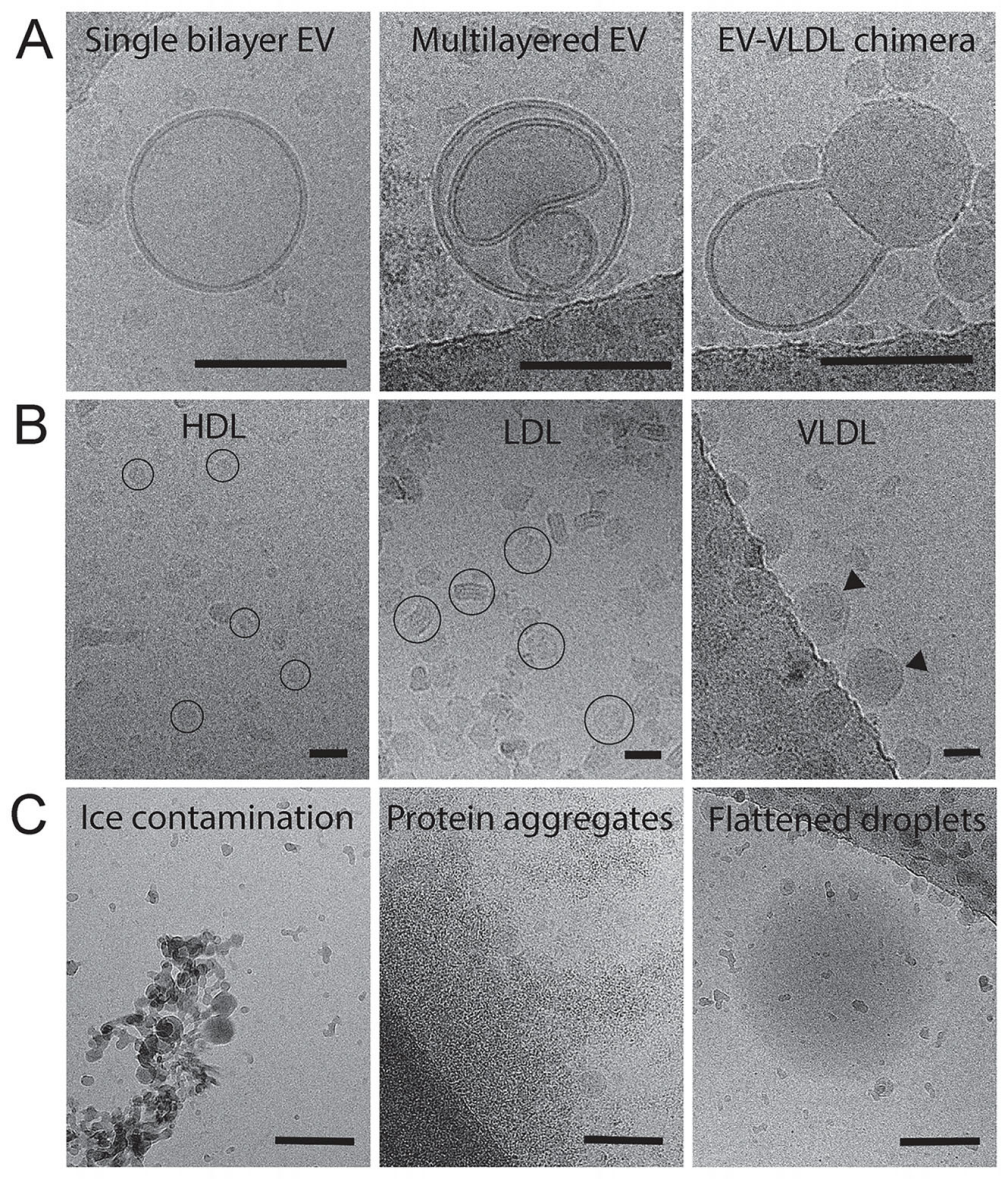
Distinct cryo‐EM structures in samples derived from human plasma, EV preparations and lipoprotein preparations. (A) Lipid bilayer‐delimited particles, including single‐bilayer EVs, multilayered EVs and EV‐VLDL chimera particles. Scale bars represent 100 nm. (B) Human lipoprotein preparations: HDL (left, examples encircled), LDL (middle, examples encircled) and VLDL (right, examples indicated by arrowheads). Scale bars represent 20 nm. (C) Other commonly observed larger structures: ice contamination, protein aggregates and flattened droplets (mainly observed in VLDL and Chylomicron samples). Scale bars represent 100 nm.

In addition to detecting EVs and small dense particles originating from conditioned medium samples, we extended our approach to assess the automated detection of non‐EV particles in compositionally heterogenous samples originating from plasma. Initial cryo‐EM observations on several plasma‐derived samples revealed the most commonly observed structures, including morphologically distinct EVs (Figure 4A), lipoproteins (Figure 4B), and other structures (Figure 4C). Next to single and multilayered EVs, also chimeras of EVs and VLDL‐like structures were observed, especially in purified VLDL samples (Figure 4A). Commercial HDL, LDL and VLDL samples (Figure 4B) generally also contained EVs. In VLDL samples (and Chylomicron preparations; data not shown) we additionally observed fuzzy electron‐dense spherical structures, which we attribute to flattened lipid‐containing droplets (Figure 4C, right panel). Furthermore, ice contamination (formed during cryo‐EM sample preparation) and protein aggregates were commonly seen (Figure 4C).

**FIGURE 5:**
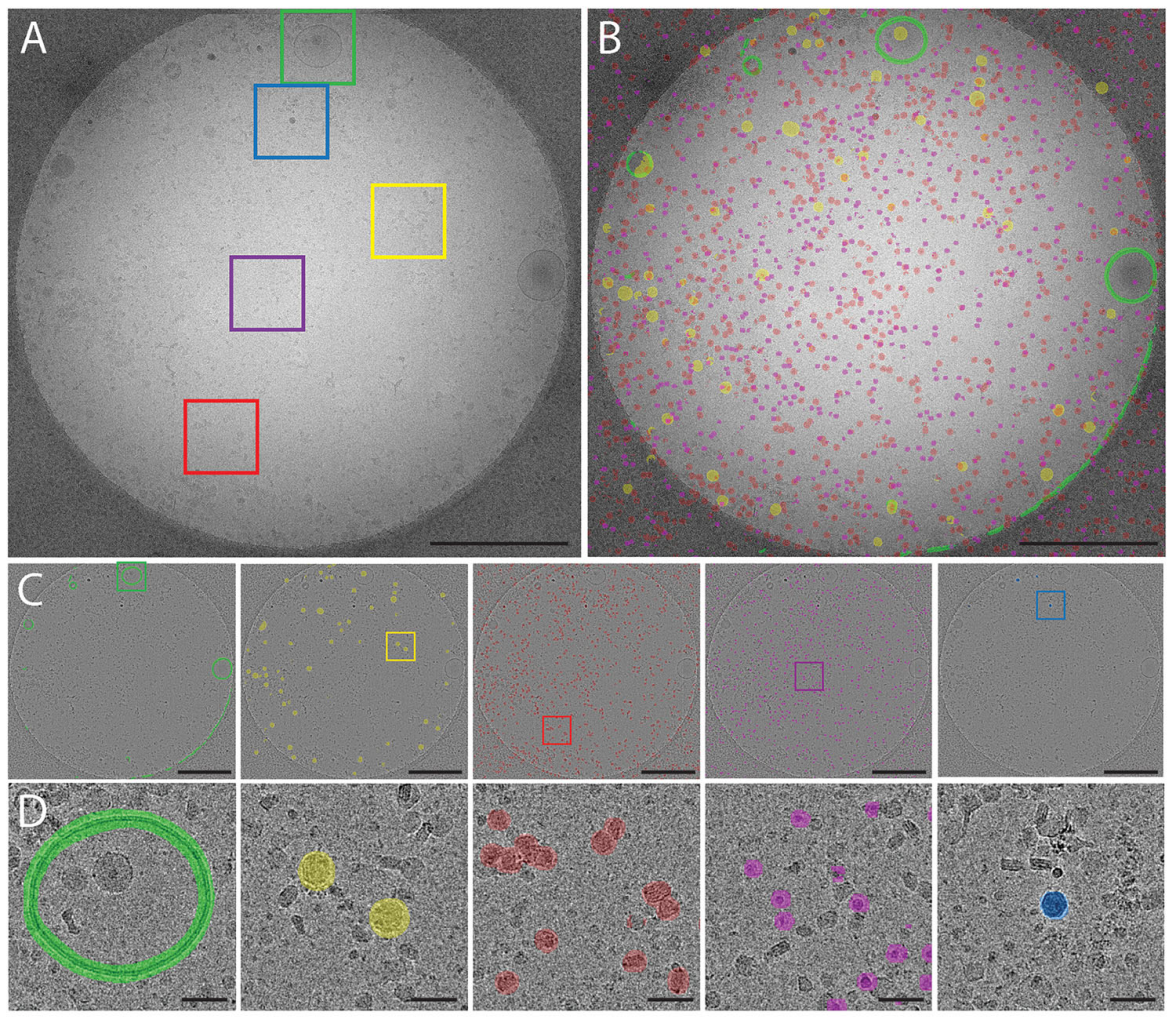
Supervised machine learning‐based detection of lipid bilayers, lipoproteins and ice contamination in EV‐lipoprotein mixtures. (A) Representative cryo‐EM image of an EV‐lipoprotein mixture. Boxes indicate the regions shown at higher magnification in (D). (B) Cryo‐EM image from A overlaid with sML predictions of lipid bilayers (green), VLDL (yellow), LDL (red), HDL (magenta) and ice contamination (blue). (C) Individual prediction masks overlaid on band‐pass‐filtered cryo‐EM images. (D) Magnified views of lipid bilayers, VLDL, LDL, HDL and ice contamination. Scale bars in A, B and C represent 500 nm, and in D represent 50 nm.

These observations guided us to annotate these structures and train CNN‐based models to predict lipoproteins, which are common confounders in blood‐derived EV samples. Additionally, we evaluated the identification of ice contamination on the surface of the thin vitreous water layer, which can resemble or obscure biological particles in cryo‐EM images. Specific models were trained to detect HDL, LDL, VLDL, and ice particles using a mixed sample of purified lipoproteins and MDA‐MB‐231‐derived EVs (Figure 5A,B).

Initial experiments showed that vitrified water thickness variations, which caused fluctuations in background contrast and brightness levels in cryo‐EM images, led to suboptimal model performance. To improve consistency in particle recognition, we applied bandpass filtering to remove background variations prior to annotation, modelling and predicting the lipoproteins and ice contamination (Figure 5C). This preprocessing step specifically improved the precise prediction of HDL, LDL and VLDL (Figure 5B–D, and Supplementary Movie 1). For EV bilayer detection, we used the previously described model trained on unfiltered liposome datasets.

A common source of false positives during bilayer detection was the rim of the holes in the carbon foil, which often resembled bilayer structures in projection (Figures 3B and 5B). However, these artifacts were excluded during downstream analysis. LDL particles were detected with minimal overlap with bilayers, ice, or HDL.

### 3.4 Detection of EVs and Lipoproteins in Plasma

**FIGURE 6:**
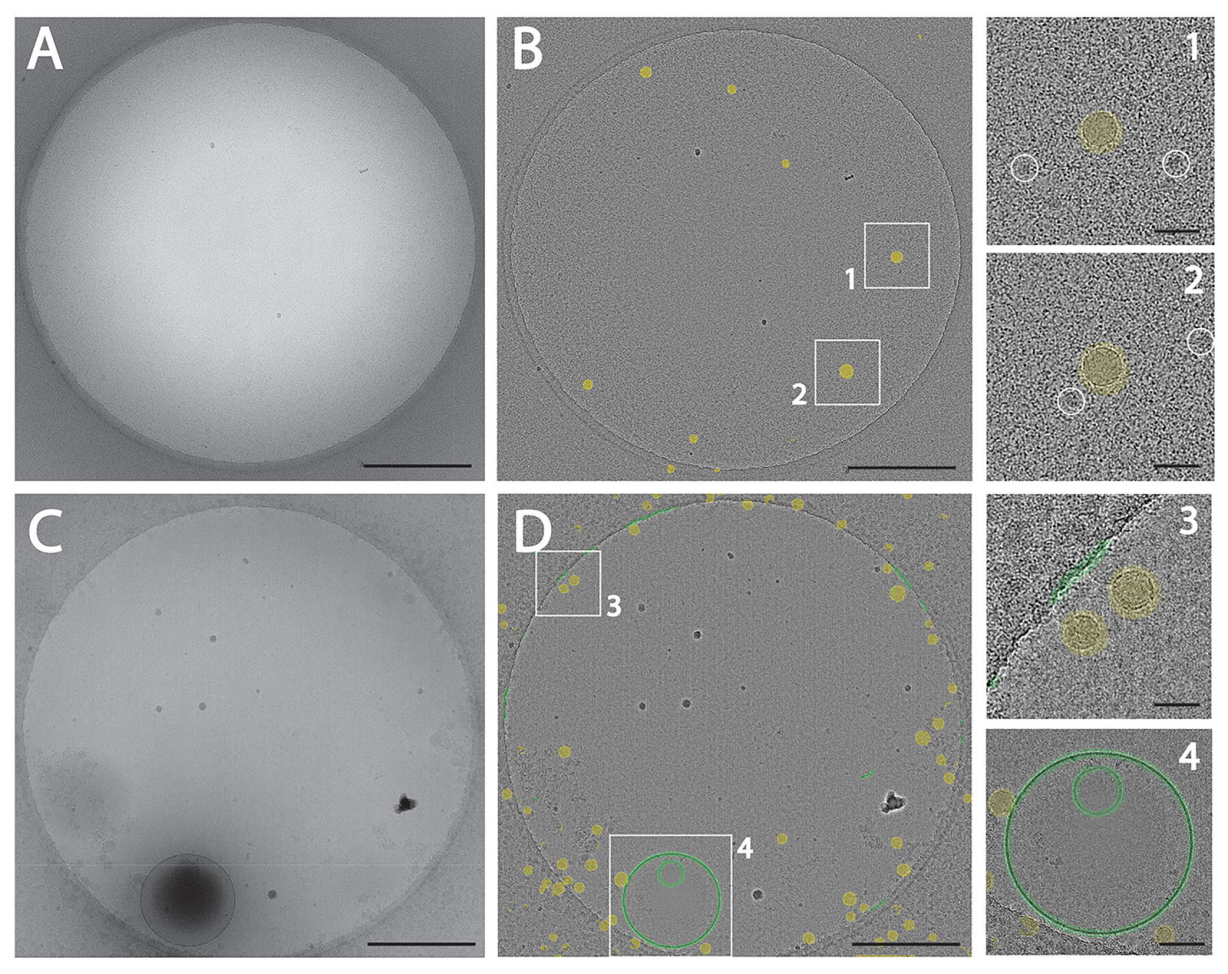
Supervised machine learning‐based detection of lipid bilayers and lipoproteins in human plasma. (A) Representative cryo‐EM image of plasma after partial removal of particles <100 kDa (B) sML predictions of very low density lipoproteins (VLDL, yellow) on band‐pass‐filtered image corresponding to A. White boxes indicate the regions shown in zooms 1 and 2. Putative low density lipoproteins (LDL) are indicated by white circles. (C) Representative cryo‐EM image of plasma processed by SEC using a 35‐nm cutoff. (D) sML predictions of lipid bilayers (green) and VLDL particles (yellow) on band‐pass‐filtered imaged corresponding to C. White boxes indicate the regions shown in zooms 3 and 4. Scale bars in A, B, C and D represent 500 nm, 25 nm in 1, 2 and 3 and 50 nm in 4.

Having demonstrated the ability to detect EVs and lipoproteins in artificially mixed samples, we next investigated whether EVs and lipoproteins could be detected in plasma using our cryo‐EM workflow. Therefore, we prepared and imaged EDTA plasma, similar as was done before (Yuana et al. 2013). Visual inspection revealed a highly granular background (Figure 6A,B) compared to the purified EV samples and EV‐lipoprotein mixtures. This background was due to the abundance of plasma proteins (albumin, immunoglobulins, fibrinogen, etc.), which obscured LDL particles, whereas VLDL particles remained discernable due to their larger size and reduced overlap within the thin vitreous ice layer. Only a limited number of small EVs (approximately 50 nm) were observed.

We applied existing sML models to these plasma images to test detection of LDL, VLDL and EV lipid bilayers. Overall, the granular background deteriorated the detection of particles, though in this plasma sample VLDL particles could be detected (Figure 6A,B). To remove the background derived from soluble proteins and small lipoproteins such as HDL and LDL, plasma was filtered by SEC using a 35‐nm cutoff. This method effectively removed the granular background enabling the detection of VLDL and EVs (Figure 6C,D).

## 4 Discussion

The EV field is rapidly advancing, yet it faces significant challenges, as outlined in the latest ISEV positioning paper MISEV 2023 (Welsh et al. 2024). One of the main challenges is the lack of standardized methods that can objectively, reproducibly, and reliably distinguish EVs from other particles, evaluate their heterogeneity in terms of size and morphology, and assess sample purity. Establishing robust analytical approaches is thereby crucial for advancing the understanding of EV biology and ensuring their effective application in both research and clinical settings.

Our study aimed to address these challenges by developing a workflow that integrates automated cryo‐EM imaging and machine learning‐assisted particle detection, enabling the objective, reproducible, and high‐throughput visualization, detection and analysis of EVs and co‐isolated non‐EV particles. Cryo‐EM imaging is one of the most accurate methods for structural imaging, size measurement, and purity assessment. We refrained from other imaging techniques, such as transmission and scanning electron microcopy, as their sample preparation methods (e.g., dehydration) compromise the structural integrity of EVs, obscure the lipid bilayer, and distort their size and appearance. Unlike methods that rely on indirect measurements or predefined assumptions for EV identification (Szatanek et al. 2017), our approach allows for the direct observation of the EV lipid bilayer, a defining characteristic according to the MISEV guidelines (Welsh et al. 2024). This capability is essential for ensuring that particles identified and characterized as EVs truly meet the structural criteria established in the field. A key strength of our approach is the ability to both image and detect the lipid bilayer in EVs, allowing for the clear distinction between EVs and other non‐EV particles lacking a lipid bilayer. This distinction is crucial for improving the purity assessments of EV samples, identification of EV‐associated structures and enhancing the reliability of downstream analyses and applications.

In addition, our study introduces a highly automated and reproducible pipeline that significantly advances the application of cryo‐EM for EV research. While, cryo‐EM is typically used to demonstrate the presence of EVs in a sample (Yuana et al. 2013; Rontogianni et al. 2019; Yoshioka et al. 2013; Zonneveld et al. 2014), it is not commonly employed for comprehensive sample assessment due to the time‐consuming nature of manual image acquisition and analysis. Hence, cryo‐EM approaches typically capture only a limited number of images, with variable quality and non‐predefined ROI selection, focusing on areas containing intact EVs (Yuana et al. 2013). As a result, acquired images may not fully reflect the content of the sample, (Rikkert et al. 2019). Thus, implementing highly automated acquisition and analysis workflows increases efficiency, ensures objective and reproducible imaging and analysis, and minimizes operator‐dependent biases, while providing a more accurate and comprehensive representation of the sample contents.

Several studies have performed extensive cryo‐EM imaging and analysis to characterize and classify EVs (Yuana et al. 2013; Arraud et al. 2014; Brisson et al. 2017; Emelyanov et al. 2020; Hoog and Lotvall 2015; Parra et al. 2024; Kapoor et al. 2023; Neyroud et al. 2022; Zabeo et al. 2017; Pernice et al. 2023; Miroshnikova et al. 2023; Conde‐Vancells et al. 2008). In some studies, (Yuana et al. 2013; Hoog and Lotvall 2015; Parra et al. 2024; Miroshnikova et al. 2023) automated image acquisition schemes were used, generally recording over 1000 images. In the other studies, between ∼100 and 800 images were recorded. Unfortunately, detailed information concerning the imaging conditions like magnification / pixel size, total electron dose and defocus values was not always reported, making it difficult to repeat the imaging conditions. Moreover, image quality varied between studies, and the lipid bilayer of the EVs was not always visible. Hence, we propose standardization of imaging settings to enable reproducibility and data comparison between studies.

After extensive imaging using reproducible and automated routines, we applied sML for particle detection and classification to analyse EV samples even at low particle concentrations. We used a 2D adaptation of a CNN architecture previously shown to be effective in detecting highly variable mitochondrial structures (Xiao et al. 2018). In principle, machine learning models can be created to detect any structurally distinct feature, including fibres (Zhang et al. 2022), proteins (Chung et al. 2022), carbon foil (Sanchez‐Garcia et al. 2020), and ice contamination (Eldar et al. 2022). By manually annotating a subpopulation of particles and training a CNN model, we created several models capable of detecting EVs, small dense particles, lipid bilayers, HDL, LDL and VLDL in cryo‐EM images. The advantage of using a model and sML prediction of structures over complete manual annotation lies in its efficiency for handling large datasets, making it especially valuable when dealing with samples having low particle concentrations. Additionally, the same model can be applied to different sample types acquired under the same imaging conditions, allowing for more objective comparisons between samples. Therefore, we also propose to generate standard models to enable reproducible detection of different particles, and to allow comparison between samples. A disadvantage is that machine learning performance metrics depend on multiple factors, including image quality, particle morphology, filtering, and the extend of manual annotation. Consequently, performance metrics should be interpreted with caution. In this study we manually corrected some prediction errors to obtain more accurate quantification and size measurement of the particles. While this improves reliability, such manual intervention is neither practical nor desirable for high‐throughput analysis of large datasets. Further methodological improvements, such as additional improved processing, artifact correction and instance‐based segmentation (instead of the current pixel‐based approach) might be required to improve the accuracy of automated quantification and size estimation.

Several studies have used cryo‐EM to assess size distributions and perform structural classification of EV particles (Yuana et al. 2013; Arraud et al. 2014; Brisson et al. 2017; Emelyanov et al. 2020; Hoog and Lotvall 2015; Parra et al. 2024; Kapoor et al. 2023; Neyroud et al. 2022; Zabeo et al. 2017; Pernice et al. 2023; Miroshnikova et al. 2023; Conde‐Vancells et al. 2008). With the exception of one (Kapoor et al. 2023), these analyses relied on manual detection, quantification, and size measurements using tools such as ImageJ/FIJI (Schindelin et al. 2012) or IMOD (Kremer et al. 1996). Across these studies, various structural features of EVs were identified, including the number of lipid bilayers (single, double, or multilamellar), electron density (associated with cargo), surface densities (reflecting membrane‐associated proteins), morphology (spherical, tubular, pleomorphic), and structural integrity (intact vs. disrupted). In addition to these commonly described features, our study detected lipoproteins and small dense particles, resembling exomeres. While these particles do not meet the formal MISEV2023 criteria for EVs, their co‐isolation and potential biological relevance highlight the importance of their recognition in EV preparations. Overall, structural classification of EVs across studies appeared incomplete and often inconsistent. Also, classification schemes include categories which are not mutually exclusive, such as “single‐layer” and “tubular” EVs. A general and uniform classification would be highly desirable to enable consistent comparison between studies.

Another major advantage of large‐scale high‐resolution cryo‐EM imaging combined with machine learning is its ability to detect and quantify not only EVs but also any structurally distinct feature within a sample, including impurities and EV‐associated structures. Here, we exemplified this by detecting lipoproteins and ice contamination. However, this approach can be extended to soluble proteins, viruses and antibody aggregates (data not shown), provided that a suitable model is trained for each specific structure. Such classification could provide valuable insights for optimizing purification strategies and isolating specific EV subtypes, enabling more detailed compositional analyses, e.g. of the protein corona, and assessing their functional roles.

Despite the advantages of the approach we developed, several challenges remain. First, access to cryo‐EM facilities and expertise may be limited in the EV field, potentially hindering its widespread adoption. Nevertheless, the increasing number of cryo‐EM facilities (Zimanyi et al. 2022), along with advances in automated imaging have made cryo‐EM more accessible to non‐experts. In this study, we used standard data acquisition schemes using widely available cryo‐EM software, ensuring that our approach can be readily available for use across different facilities. Sharing data collection parameter files further facilitates consistent imaging and reproducibility. Moreover, facilities using alternative acquisition software (Schorb et al. 2019; Sawh‐Gopal et al. 2023) can easily replicate our imaging settings. Here, we also show that EV particle analysis can be performed in a reproducible and broadly accessible manner using open‐source software such as ImageJ/FIJI, (Schindelin et al. 2012) for calculating Hough transforms. It is worth noting that several other analysis packages are also available (Hermann et al. 2014; van Buren et al. 2023).

The second challenge was prediction performance and quality assessment of our models. Since sML relies on manually annotated ground truth data, the performance of the model can be influenced by the quantity and structural variability of the training data. The limited training set in this study may have constrained model generalization. Expanding the dataset in future work will improve the accuracy and robustness of predictions across different EV sources, such as plasma and urine. Furthermore, we did not extensively test different CNN networks, optimize our CNN models, implemented pre‐filtering of data, used non‐maximum suppression algorithms to prevent overlapping model predictions, so there are several opportunities to improve the quality of the results in the future. Therefore, we did not extensively evaluated the model performances using standard performance metrics. Nevertheless, we have demonstrated the feasibility of reproducibly and automatically detecting and analysing EVs and other particles in large datasets, highlighting its potential for objective purity assessment in EV samples.

Additional challenges arise from cryo‐EM sample preparation‐induced artifacts. For instance, particles may adhere preferentially to the carbon surface of the support grid (Snijder et al. 2017; Tonggu and Wang 2020), potentially biasing their distribution within the vitrified sample. In addition, well‐document issues such as preferential orientation and denaturation of (membrane) proteins at the air‐water interface (D'Imprima et al. 2019; Drulyte et al. 2018) may similarly affect the structural integrity of EVs. Another issue arises from the compression of large EVs when the thickness of the vitreous ice, typically 50–200 nm, is insufficient to accommodate their full size. This phenomenon may particularly impact the accuracy of size measurements for EVs >200 nm. Overall, large scale cryo‐EM imaging and sML alone do not necessarily provide an accurate quantitative description of all sample components, but can detect the structural variability of EVs and presence of sparse structures that might remain undetected by other techniques.

Other cryo‐EM techniques can provide deeper insight into the structure‐function relationships of the different EV classes. For instance, cryo‐electron tomography (cryo‐ET) offers detailed 3D structural information, including the presence and localization of proteins and genomic material within or associated at the outside of EVs (Hoog and Lotvall 2015; Chaya et al. 2024; Lozano‐Andrés et al. 2023), and combination with cryo‐correlative light and electron microscopy (cryo‐CLEM) (Linares et al. 2015; Linares et al. 2017; Metskas and Briggs 2019) or antibody‐gold labelling techniques enable the identification of specific proteins and their localization with specific EVs. These advanced methods will significantly improve the understanding of EV structure and function.

In conclusion, our study presents a comprehensive and reproducible approach to address critical challenges in the EV field. By integrating automated cryo‐EM imaging, sML, and particle analysis, we provide a robust workflow that enables the reliable detection and classification of EVs and non‐EV particles, distinguishing them based on key structural features. Our approach offers a valuable addition to currently existing EV sample analysis techniques, and can specifically impact sample purity assessments and characterization.

## Author Contributions

**Agustin Enciso‐Martinez**: conceptualization, writing – original draft, writing – review and editing, investigation, funding acquisition, project administration, resources, validation. **Frank G.A. Faas**: software, formal analysis, data curation, resources. **Anja W.M. de Jong**: investigation, data curation. **Ton G. van Leeuwen**: resources, supervision. **Rienk Nieuwland**: supervision, writing – review and editing. **Edwin van der Pol**: supervision. **Peter ten Dijke**: writing – review and editing, supervision, funding acquisition, resources. **Roman I. Koning**: conceptualization, investigation, writing – original draft, methodology, visualization, writing – review and editing, software, project administration, supervision, data curation, formal analysis, resources, validation.

## Conflicts of Interest

E.v.d.P. is co‐founder and shareholder of Exometry B.V., Amsterdam, The Netherlands.

## Supporting information




**Supporting Information**: jev270273‐sup‐0001‐SuppMat.docx

**Supporting Information**: jev270273‐sup‐0002‐EV.mp4

## Data Availability

The data that support the findings of this study are available from the corresponding authors upon reasonable request.
