# Supplementary material for: Automated Cryo‐EM and Supervised Machine Learning Enable Reproducible Characterization of Extracellular Vesicles and Co‐Isolating Particles
Source: J Extracell Vesicles. 2026 Apr 14;15(4):e70273. doi: 10.1002/jev2.70273 (PMC13077556; doi:10.1002/jev2.70273)
Supplement: Supplementary file 1 — Supporting Information: jev270273‐sup‐0001‐SuppMat.docx [file JEV2-15-e70273-s002.docx]

## Supplementary data

### Immunoblotting of EV lysate for tetraspanin markers

To provide orthogonal validation of EV identity beyond cryo-EM morphology, we performed dot immunoblotting for canonical EV tetraspanin markers, i.e., CD9 and CD63 on EVs isolated from MDA-MB-231 conditioned media. MDA-MB-231 cells were cultured and conditioned medium collected and processed for EV isolation as described in Materials and Methods (*Cell Culture and EV collection* and *EV purification)*. The EV-containing medium was mixed at a 1:1 volume ratio with lysis buffer (9% [w/v] glycerol, 3% [w/v] Sodium Dodecyl Sulfate (SDS), and 0.2 M Tris-HCl, pH 6.8). Next, 0.22 μm pore size nitrocellulose membranes were spotted with 5 µL of sample, allowed to dry and re-spotted with the same volume of sample. The membranes were blocked with 5% (w/v) non-fat milk for 1 hour at room temperature in a shaker, followed by washing twice with TBST buffer (20 mM Tris-HCl, pH 7.6, 150 mm NaCl, and 0.1% [v/v] Tween 20). The membranes were incubated with primary antibodies anti-human CD9 (HI9a, Cat. No. 312102, Biolegend, 1:1000) and anti-human CD63 (MX-49.129.5, Cat. No. sc-5275, Santa Cruz, 1:1000) 12 h at 4 °C. Antibodies were diluted in 3% bovine serum albumin (BSA) with 0.02% (w/v) NaN_3_. After overnight incubation, the membranes were 4x washed with TBST for 5 min each time in a shaker. Next, the membranes were incubated with secondary antibodies anti-mouse (1:10000 in 5% [w/v] non-fat milk) for 1 h in a shaker. The membranes were washed 3x with TBST for 5 min each time. The membranes were dried and enhanced chemiluminescent (ECL) substrate (Carity Western, Cat. No. 1705061, Bio-Rad) was loaded on the membranes, covered with a plastic foil and detected using a Bio-Rad ChemiDoc imaging system.


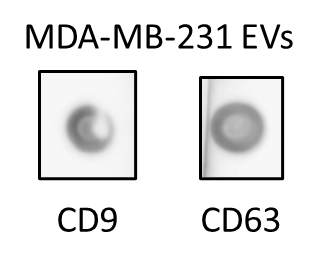


##### Figure S1. Immunoblotting of lysates from MDA-MB-231-derived EVs using antibodies against the tetraspanins CD9 and CD63.

### Orthogonal biochemical validation of EV presence by density gradient ultracentrifugation and dot blot analysis

*Density gradient ultracentrifugation.* To complement cryo-EM-based structural identification, EVs were validated using density gradient ultracentrifugation followed by immunochemical analysis. Density gradient ultracentrifugation was performed largely as described by X. Zhang et al. {Zhang, 2020 #132}, with minor modifications. Briefly, 1 mL of EV-containing sample was layered onto a discontinuous 10-50% iodixanol gradient (OptiPrep^TM^, Cat. No. 07820, StemCell). Gradients were centrifuged in polypropylene SW40 tubes (Cat. No. 331374, Beckman Coulter) for 16 h at 160,000 × g at 4 °C using an SW40Ti rotor in an ultracentrifuge (Optima XE-90, Beckman Coulter). Fractions (1 mL) were collected from the top of the gradient and diluted with PBS containing 5 mM EDTA to reduce viscosity. To determine the absolute density of each fraction, density measurements were performed using a density meter (DMA - 38, Anton Paar).

*Protein precipitation and dot blot analysis.* Proteins from density gradient fractions were precipitated using trichloroacetic acid in the presence of sodium deoxycholate (DOC), as described by Zhang et al. {Zhang, 2020 #132}. Precipitated proteins were resuspended in lysis buffer (9% [w/v] glycerol, 3% [w/v] Sodium Dodecyl Sulfate (SDS), and 0.2 M Tris-HCl, pH 6.8) and analyzed by dot blot, with 8 µL of each fraction spotted on membranes to assess the distribution of EV-associated markers across the gradient


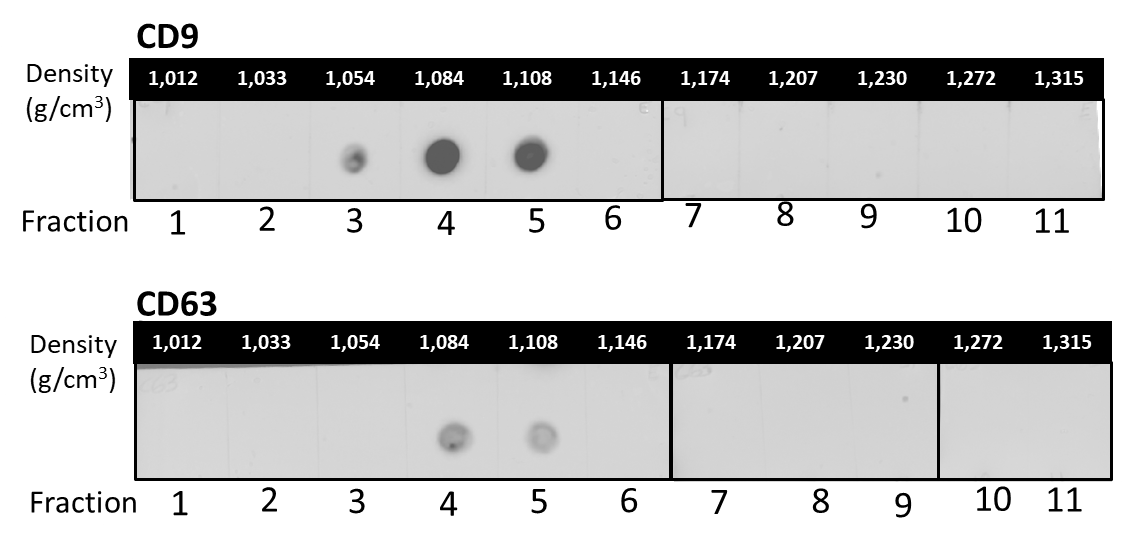


##### Figure S2. Dot blot analysis of MDA-MB-231-derived EVs following density gradient ultracentrifugation and protein precipitation. Eleven fractions collected from the top of the OptiPrep gradient were analyzed by dot blot using antibodies against the EV markers CD9 and CD63.

### Immunoblotting for ApoA1 and ApoB100 of lysates from lipoprotein-containing samples

To biochemically confirm the presence and identity of lipoproteins in the commercial preparations, immunoblotting was performed using antibodies against apolipoprotein A-I (ApoA-I) and apolipoprotein B100 (ApoB100). Dot blot analysis was performed on commercial high-density lipoproteins (HDL, 14.1 mg/mL, Cat. No.: LP3-5MG, EMD Millipore Corp.) and low-density lipoproteins (LDL, 6.56 mg/mL, Cat. No.: 437644-10MG, EMD Millipore Corp.) preparations using the procedure described above for EV-containing samples (see *Immunoblotting of EV lysate for tetraspanin markers*). Membranes were incubated with a primary antibody against human apolipoprotein A-I (ApoA-I, Cat. No. sc-376818, Santa Cruz Biotechnology, 1:1000 dilution; HDL marker), or human apolipoprotein B100 (ApoB100, Cat. No. AF3260, Biotechne, R&D Systems, 1:1000 dilution; LDL marker), followed by species appropriate secondary antibodies: anti-mouse (ApoA-I, 1:10000 dilution) or anti-goat (ApoB100, 1:5000 dilution).

**
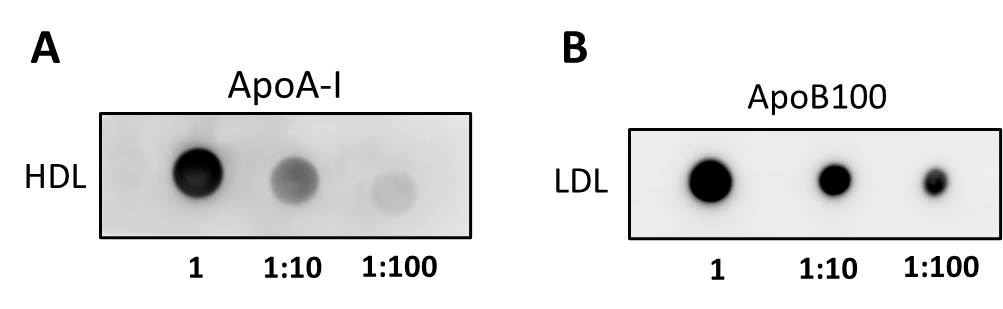
**

Figure S3. Immunoblot analysis of high-density lipoprotein (HDL) and low-density lipoprotein (LDL) preparations using antibodies against apolipoprotein A-I (ApoA-I) and apolipoprotein B100 (ApoB100), respectively. Serial dilutions of each sample (undiluted stock, 10×, and 100× dilutions) were spotted onto the membranes.

### Orthogonal biochemical validation of lipoprotein presence by density gradient ultracentrifugation and dot blot analysis

To provide orthogonal biochemical validation of lipoprotein presence, density gradient ultracentrifugation followed by dot blot analysis was performed on lipoprotein-containing samples. Lipoprotein samples were processed using the same density gradient ultracentrifugation and dot blot workflow described above (see *Orthogonal biochemical validation of EV presence by density gradient ultracentrifugation and dot blot analysis*), with the exception that protein precipitation was not performed. Briefly, purified human low-density lipoproteins (LDL, 6.56 mg/mL, Cat. No.: 437644-10MG, EMD Millipore Corp.), very-low-density lipoproteins (VLDL, 0.8 mg/mL, Cat. No.: 437647-5MG, Calbiochem), and chylomicrons (Cat. No.: SRP6304, Sigma-Aldrich) were pooled and diluted in PBS to a final volume of 1 mL by adding 5 µL of each stock solution. The pooled lipoprotein mixture was subjected to density gradient ultracentrifugation followed by dot blot analysis. The analysis focused on the detection of apolipoprotein B100 (ApoB100), which is present on VLDL and LDL particles.


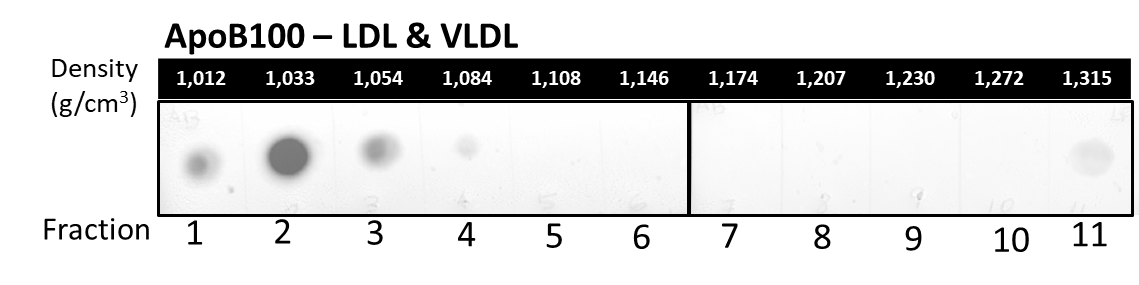


##### Figure S4. Dot blot analysis of lipoproteins samples following density gradient ultracentrifugation. Eleven fractions collected from the top of the OptiPrep gradient were analyzed by dot blot using antibodies against LDL and VLDL marker ApoB100.

### Cryo-EM image of commercial VLDL

##### VLDL particles strongly attach to the surface of the carbon. Vesicles and flattened chylomicrons (dark blobs) are observed. Width of top image is 2 micron of bottom image 1 micron.


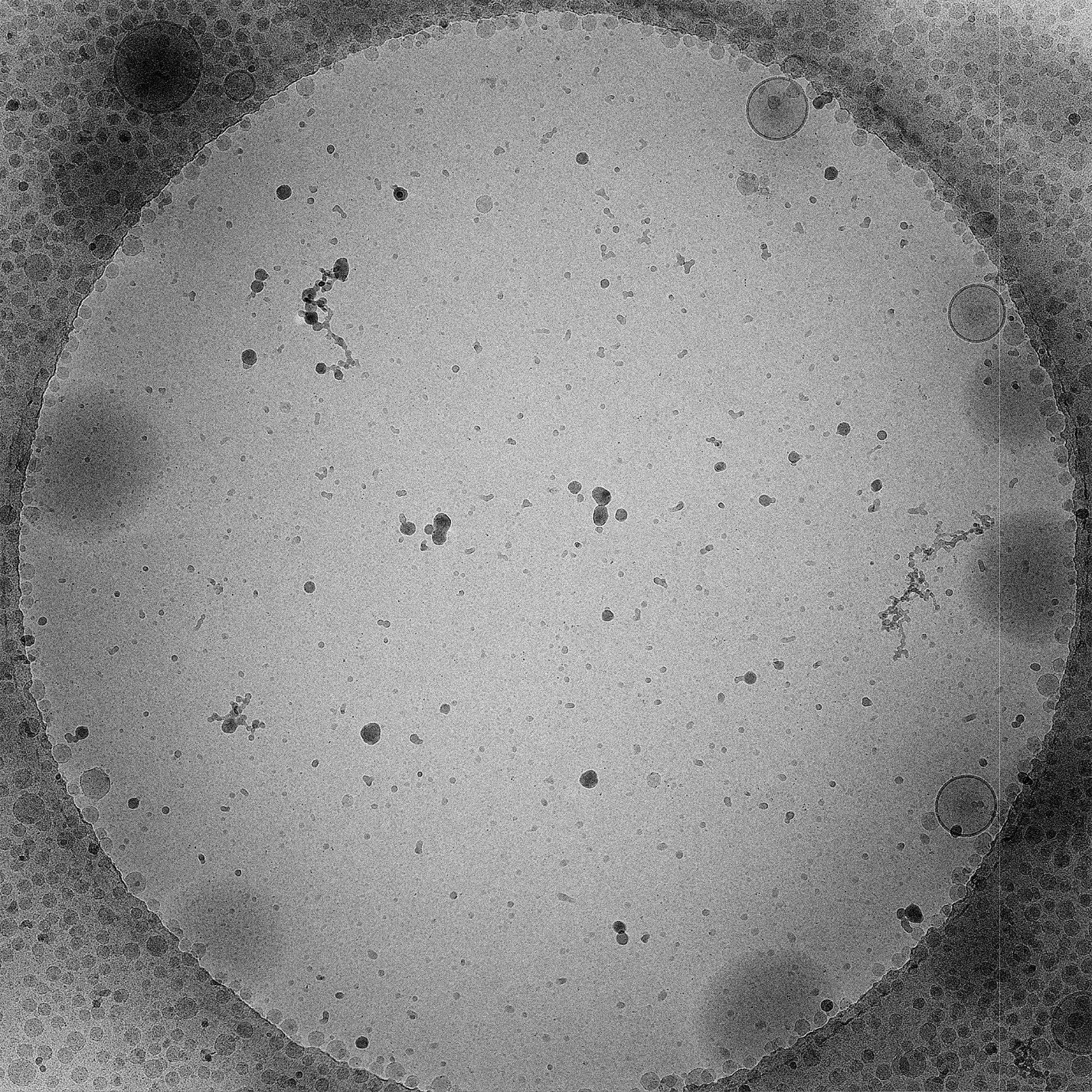


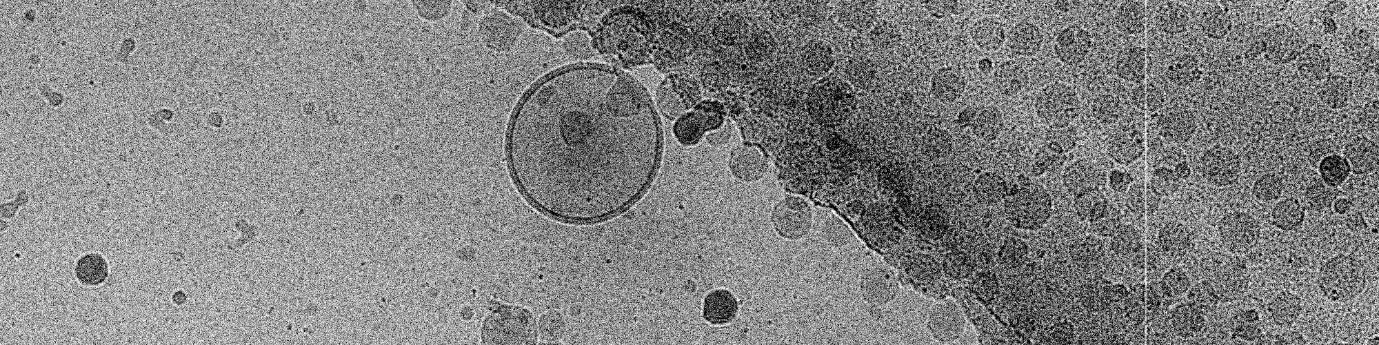


### Cryo-EM image of commercial LDL

##### Many LDL particles are present in top and side view (exhibiting typical lining). EVs are only sporadically present. Width of top image is 2 micron of bottom image 1 micron.


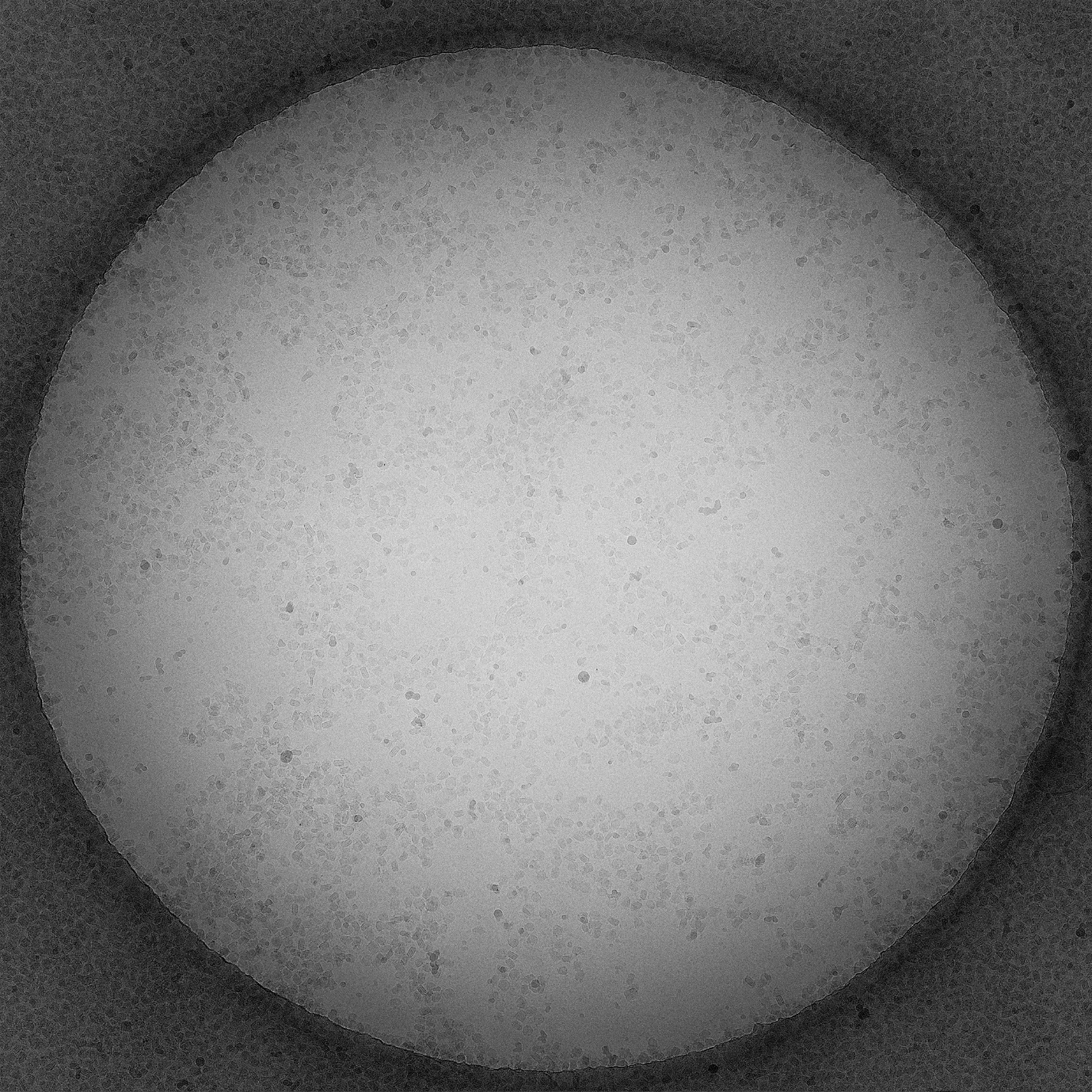


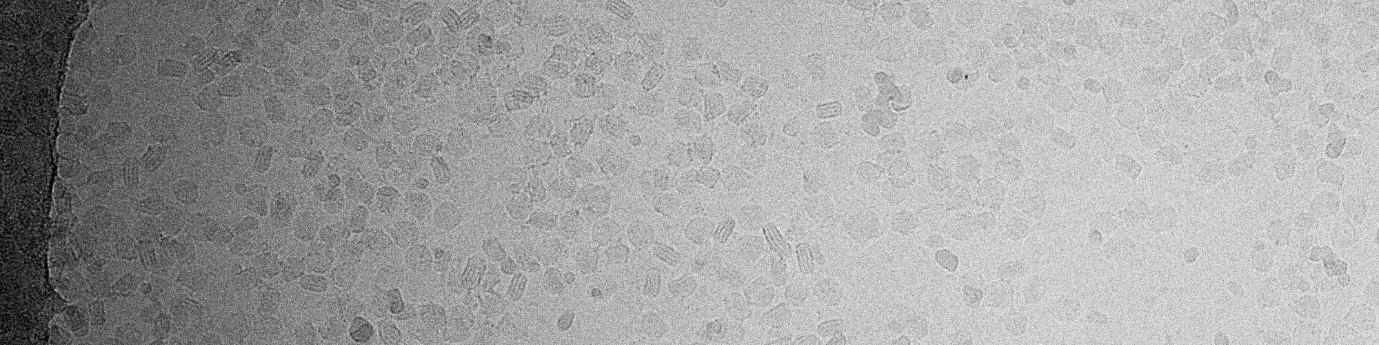


### Cryo-EM image of commercial HDL

##### HDL particles and aggregated HDL can be observed. HDL is difficult to detect due to its small size and the presence of ice contamination. Occasionally VLDL and vesicles are present in the images. Width of top image is 2 micron of bottom image 1 micron.


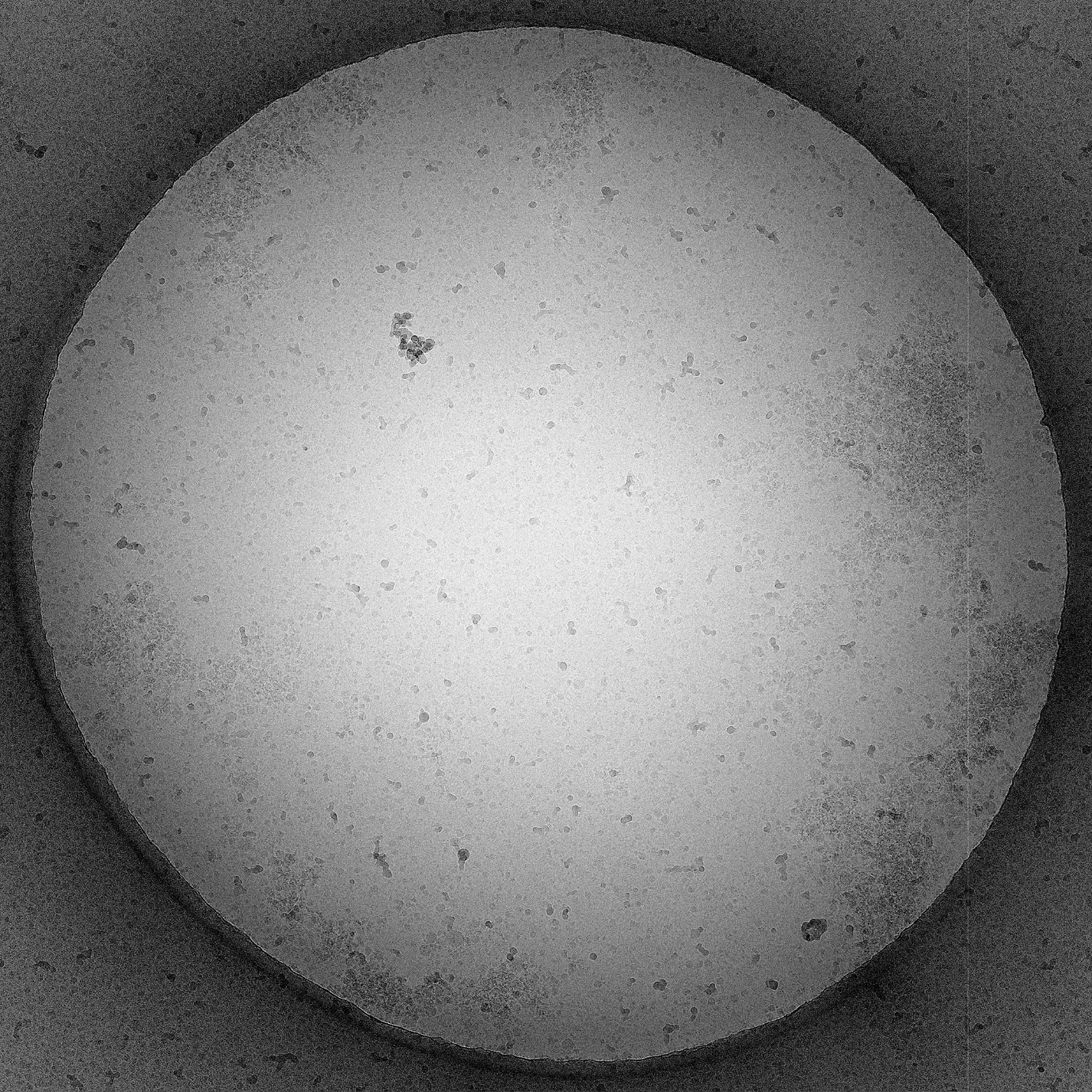


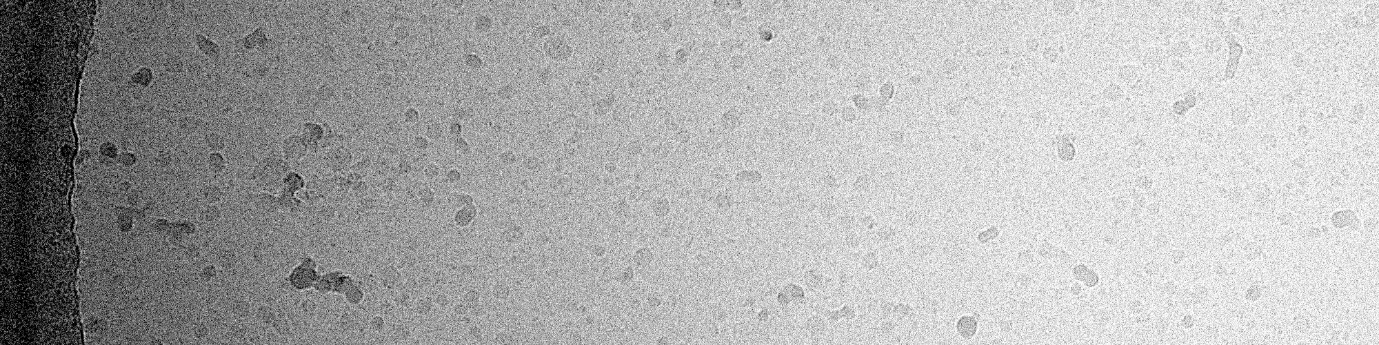


#####

### Processing of EV particles from EM images using supervised machine learning


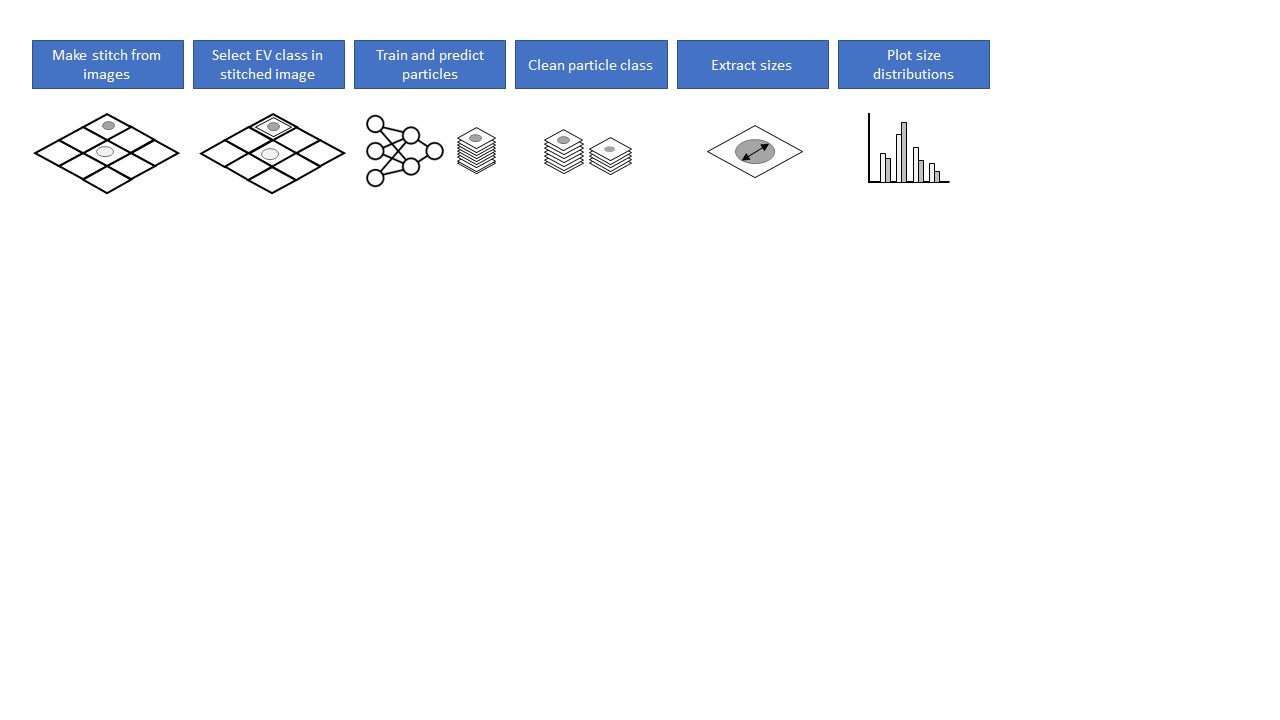


Supervised machine learning is performed via a custom software interface (myCAVIA; manuscript in preparation). In short:

1. Make composite image from all recorded cryo-EM images
2. Manual annotation of particles per class:
   - Manually annotate a limited number of particles
   - Add selection boxes around annotated particles
   - Make trainingset of all boxes and annotations
3. Train models
   - Select set and train CNN model using Keras/TensorFlow
   - Do for all separate classes
4. Predict particles
   - Select model and predict particle in original image
5. Improve predictions and clean particle class
   - If necessary, add new annotations and boxes and repeat model training and predictions
   - Manually clean predictions by removing false positive artifacts
6. Extract data and plot size
   - Export surface area from predictions to .cvs and plot size distributions in Excel

### Hough circle transforms using Fiji

Input for Hough circle transformations are the stack of the segmentations

1. Open dataset in Fiji
2. Prepare segmentation dataset
   1. For circles (whole particle predictions):
      1. Process – find edges
   2. For rings (bilayer predictions):
      1. Process – Binary – Make binary
      2. Process – Binary – Skeletonize

(optional to remove false positives based on size and shape:)

- - 1. Analyze – Analyze particles
       1. Size: 10 – infinity (to remove small islands)
       2. Circularity: 0.00 – 1.00
       3. Show: masks
       4. Check: clear results
    2. Process – Binary - Dilate

1. Open Hough circle Transform plugin
   1. Plugins – UCB vision Sciences – Hough circle transform
   2. Set advances mode and test settings: (bin3 on circles :)
      1. Minimum radius: 6
      2. Maximum radius: 100
      3. Radius search increment 1
      4. Number of circles found 1024
      5. Hough threshold 0.8
      6. Transform resolution 100
      7. Clear neighborhood ratio 10
   3. Set advances mode and test settings: (bin3 on rings:)
      1. Minimum radius: 6
      2. Maximum radius: 100
      3. Radius search increment 1
      4. Number of circles found 1024
      5. Hough threshold 0.5
      6. Transform resolution 100
      7. Clear neighborhood ratio 10
2. Tick export measurements to results table

### EPU settings file

The settings file is used in EPU with all image acquisition settings.

EPU_EV_110_5600_15000_49000_RIK_7_ZLP.sxml (52 kb)
